# Bioaccessibility, Intestinal Permeability and Plasma Stability of Isorhamnetin Glycosides from *Opuntia ficus-indica* (L.)

**DOI:** 10.3390/ijms18081816

**Published:** 2017-08-22

**Authors:** Marilena Antunes-Ricardo, César Rodríguez-Rodríguez, Janet A. Gutiérrez-Uribe, Eduardo Cepeda-Cañedo, Sergio O. Serna-Saldívar

**Affiliations:** Tecnologico de Monterrey, Centro de Biotecnología-FEMSA, Escuela de Ingeniería y Ciencias, Av. Eugenio Garza Sada 2501 Sur, Monterrey 64849, Mexico; marilena.antunes@itesm.mx (M.A.-R.); cesarr33@gmail.com (C.R.-R.); jagu@itesm.mx (J.A.G.-U.); eduardo.cepedac@outlook.com (E.C.-C.)

**Keywords:** bioaccessibility, permeability, glycosides, isorhamnetin, flavonoids, *Opuntia ficus-indica*

## Abstract

Isorhamnetin glycosides are representative compounds of *Opuntia ficus-indica* that possess different biological activities. There is slight information about the changes in bioaccessibility induced by the glycosylation pattern of flavonoids, particularly for isorhamnetin. In this study, the bioaccessibility and permeability of isorhamnetin glycosides extracted from *O. ficus-indica* were contrasted with an isorhamnetin standard. Also, the plasma stability of these isorhamnetin glycosides after intravenous administration in rats was evaluated. Recoveries of isorhamnetin after oral and gastric digestion were lower than that observed for its glycosides. After intestinal digestion, isorhamnetin glycosides recoveries were reduced to less than 81.0%. The apparent permeability coefficient from apical (AP) to basolateral (BL) direction (Papp_(AP-BL)_) of isorhamnetin was 2.6 to 4.6-fold higher than those obtained for its glycosides. Isorhamnetin diglycosides showed higher Papp_(AP-BL)_ values than triglycosides. Sugar substituents affected the Papp_(AP-BL)_ of the triglycosides. Isorhamnetin glycosides were better retained in the circulatory system than the aglycone. After intravenous dose of the isorhamnetin standard, the elimination half-life was 0.64 h but increased to 1.08 h when the *O. ficus-indica* extract was administered. These results suggest that isorhamnetin glycosides naturally found in *O. ficus-indica* could be a controlled delivery system to maintain a constant plasmatic concentration of this important flavonoid to exert its biological effects in vivo.

## 1. Introduction

The most abundant flavonoid in *O. ficus-indica* (L.) Mill is isorhamnetin and it is found as mono-, di-, and tri-glycosides [1,2,3,4,5]. Isorhamnetin and its glycosides possess effects in the adipogenesis inhibition, body weight reduction, ameliorate insulin resistance, and alleviate hepatic steatosis in diet-induced obese mice via the suppression of PPARγ activity [6,7,8,9,10,11]. Moreover, these flavonoids have shown anti-inflammatory and chemopreventive activities [12,13,14,15]. Since these compounds have potential benefits on health, more research is required to incorporate them in new food products or as bioactive ingredients for natural drugs.

In general, flavonoids aglycones have shown a poor bioavailability both in vitro and in vivo due to their limited ability to pass across the lipid rich biological membranes [16,17,18]. Flavonoid glycosides have a higher accessibility than their corresponding aglycones after simulated digestion, probably due to their improved aqueous solubility and their higher stability during digestion. Isorhamnetin aglycone from almond skins showed a lower bioaccessibility (25.1 ± 7.0%) than isorhamnetin monoglycoside (93.2 ± 0.2%) and diglycoside (66.8 ± 1.7%) after a simulated digestion [19]. Precisely, digestion allows the release of the compounds from the food matrix in the gastrointestinal lumen, making them available for absorption; this has been defined as bioaccessibility [20].

On the other hand, flavonoids have shown an effective intestinal absorption across the Caco-2 cell monolayer by passive diffusion; however, a variety of flavonoid conjugates, like esters and glycosides, showed additional mechanisms which increase their absorption [21,22,23]. In the case of isorhamnetin, both transcellular and paracellular transport pathways have been reported through different cell transporters like the permeability glycoprotein (P-gp), the breast cancer resistance protein (BCRP), and the multidrug resistance-associated protein 2 (MRP2) [24]. Quercetin undergoes intestinal and/or hepatic glucuronidation and is pumped back by BCRP in the form of glucuronidated metabolites [25]. Likewise, Duan et al. [24] showed that isorhamnetin efflux was mediated by transporters such as P-gp, BCRP, and, especially, MRP2, which are vital for isorhamnetin transport in the intestine.

A strong correlation (R: 0.95) between in vitro permeability behavior of different compounds tested in the Caco-2 cell monolayer systems and their in vivo absorption have been observed [26,27]. Yasuda et al. [28] demonstrated that luteolin and its monoglucoside were quickly absorbed after administration of the *C. morifolium* flower extract to rats and differentiated Caco-2 cells. In vivo flavonoid bioavailability can differ among food sources depending on their glyosidic profile [29]. Burak et al. [30] demonstrated that the systemic availability and the maximum plasma concentration (C_max_) of quercetin was 4.8 and 5.4 times higher after ingestion of the onion skin extract than after ingestion of pure quercetin dihydrate. Hollman et al. [31] reported that quercetin glucosides from onions were better absorbed than quercetin rutinosides from apples. Furthermore, hesperetin-7-*O*-glucoside have shown a bioavailability 76% higher than hesperetin-7-*O*-rutinoside after being perfused into the proximal human jejunum. Likewise, hesperetin-7-*O*-rutinoside showed a delayed absorption after oral consumption (t_max_: 4.8 ± 2.2 h) compared to hesperetin-7-*O*-glucoside (t_max_: 0.5 ± 0.1 h) [32]. The metabolism of flavonoids has also been affected by their glycosylation pattern, as shown by Makino et al. [33] and Dueñas et al. [34], who demonstrated that quercetin glycosides maintain higher levels in plasma and have a longer residence time in the blood than quercetin aglycone.

The aim of this study was to compare the in vitro bioaccessibility and permeability of isorhamnetin and its glycosides extracted from *O. ficus-indica* after a simulated digestion using a Caco-2/HT-29 cell co-culture model. Additionally, the stability in plasma of these compounds after the intravenous administration in rats of an *O. ficus-indica* extract or isorhamnetin standard was evaluated.

## 2. Results

### 2.1. Analysis of Isorhamnetin Glycosides by High Performance Liquid Chromatography (HPLC)

**Figure 1:**
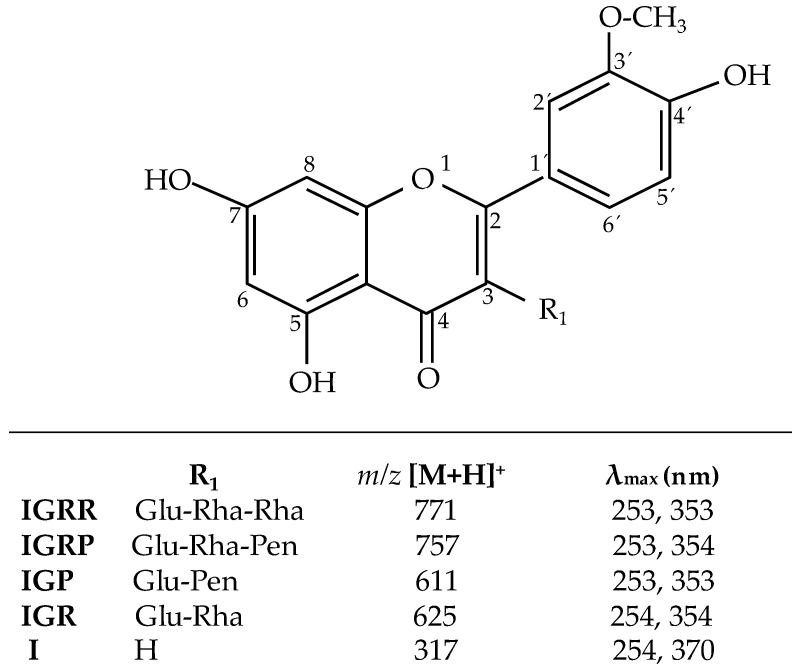
Chemical structures of isorhamnetin glycosides found in *O. ficus-indica* extract. Glc = glucose; Rha = rhamnose; Pen = pentose.

Four isorhamnetin glycosides (IGRR (isorhamnetin-3-*O*-glucosyl-rhamnosyl-rhamnoside), IGRP (isorhamnetin-3-*O*-glucosyl-rhamnosyl-pentoside), IGP (isorhamnetin-3-*O*-glucosyl-pentoside), and IGR (isorhamnetin-3-*O*-glucosyl-rhamnoside)) were identified as the most abundant flavonols in the *O. ficus-indica* extract at 365 nm (Figure 1), as previously reported by Antunes-Ricardo et al. [2] and Rodríguez-Rodríguez et al. [10] Triglycosides, IGRR ([M + H]^+^ at *m*/*z* 771) and IGRP ([M + H]^+^ at *m*/*z* 757), were identified as isorhamnetin-3-*O*-glucosyl-rhamnosyl-rhamnoside and isorhamnetin-3-*O*-glucosyl-rhamnosyl-pentoside, respectively. Likewise, the diglycosides, IGP ([M + H]^+^ at *m*/*z* 611) and IGR ([M + H]^+^ at *m*/*z* 625), were identified as isorhamnetin-3-*O*-glucosyl-pentoside and isorhamnetin-3-*O*-glucosyl-rhamnoside, respectively. Fragment showed at *m*/*z* 317 ([M + H]^+^ was identified as isorhamnetin aglycone [I].

### 2.2. Bioaccessibility of Isorhamnetin Glycosides

Isorhamnetin glycosides showed recovery percentages above 93.5% after oral and gastric phases (Table 1). However, at the end of the intestinal phase, isorhamnetin glycosides recoveries were reduced to less than 81.0% and no differences were observed between isorhamnetin and its glycosides. Regarding isorhamnetin aglycone, it showed recoveries of 35.0% and 49.0% after oral and gastric digestion, respectively. Interestingly, after intestinal digestion, its recovery increased to 74.3% since the pH was above the reported for isorhamnetin pKa (6.3), favoring its solubility (Duan et al. [24]).

### 2.3. Permeability Experiments

**Figure 2:**
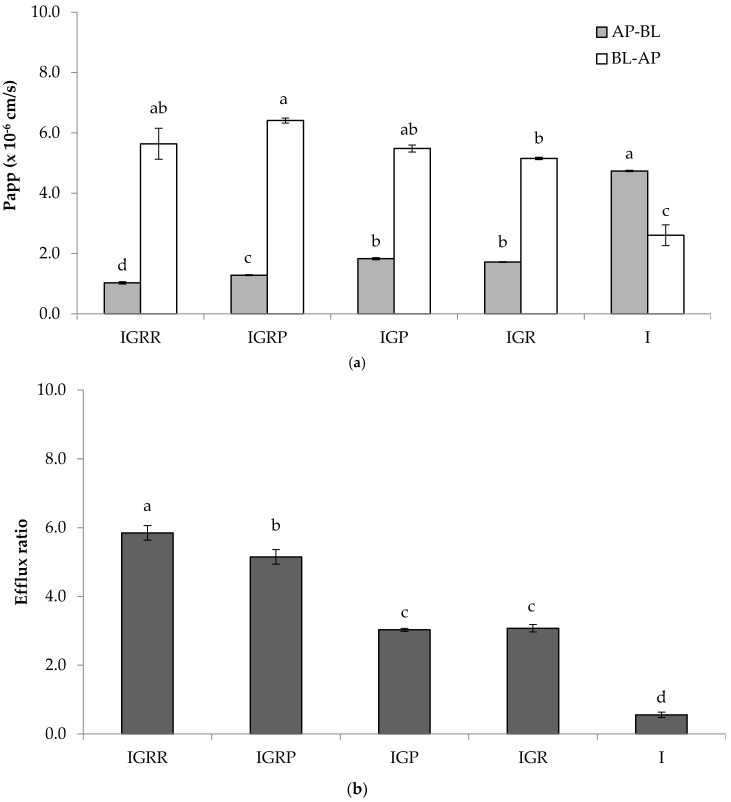
Comparison between permeability of isorhamnetin glycosides from *O. ficus-indica* extract and isorhamnetin standard in a Caco-2/HT-29 cell monolayer. (**a**) Apparent permeability (Papp) of isorhamnetin glycosides (IGRR, IGRP, IGP, IGR) and isorhamnetin standard from apical to basolateral (AP-BL), and basolateral to apical compartment (BL-AP); (**b**) efflux ratio of isorhamnetin glycosides (IGRR, IGRP, IGP, IGR) and isorhamnetin standard. Data was expressed as mean ± standard deviation. IGRR: isorhamnetin-3-*O*-glucosyl-rhamnosyl-rhamnoside; IGRP: isorhamnetin-3-*O*-glucosyl-rhamnosyl-pentoside; IGP: isorhamnetin-3-*O*-glucosyl-pentoside; IGR: isorhamnetin-3-*O*-glucosyl-rhamnoside; I: isorhamnetin. Different letters indicate significant differences among different isorhamnetin glycosides. Efflux ratio was calculated by the relation between Papp_(BL-AP)_/Papp_(AP-BL)_.

Isorhamnetin glycosides showed apparent permeability coefficients from the apical (AP) to basolateral (BL) direction (Papp_(AP-BL)_) between 1.0 and 1.8 × 10^−6^ cm/s (Figure 2a). Particularly, the Papp_(AP-BL)_ value of isorhamnetin-3-*O*-glucosyl-rhamnoside (IGR) using the Caco-2/HT-29 co-culture model was 1.72 ± 0.01 × 10^−6^ cm/s whereas the reported by Tian et al. [23] for this compound across the Caco-2 monolayers was 0.29 × 10^−6^ cm/s. This effect was more remarkable in isorhamnetin aglycone, which showed a Papp_(AP-BL)_ value of 4.74 ± 0.02 × 10^−6^ cm/s whereas Lan et al. [35] reported a Papp_(AP-BL)_ value of 2.28 ± 0.09 × 10^−6^ cm/s across the Caco-2 cells.

The Papp_(AP-BL)_ value of isorhamnetin [I] aglycone was 2.6–4.6-fold higher than the Papp_(AP-BL)_ value of isorhamnetin glycosides (Figure 2a) due to their difference in hydrophilicity that hinders their entry through the cell membrane. On the other hand, isorhamnetin diglycosides showed higher Papp_(AP-BL)_ values than triglycosides. Among isorhamnetin triglycosides, isorhamnetin-3-*O*-glucosyl-rhamnosyl-pentoside [IGRP] (1.28 ± 0.02 × 10^−6^ cm/s) showed better membrane permeability than isorhamnetin-3-*O*-glucosyl-rhamnosyl-rhamnoside [IGRR] (1.03 ± 0.04 × 10^−6^ cm/s).

Regarding the apparent permeability from the basolateral (BL) to the apical (AP) direction, it was 3.0–5.8-fold higher for isorhamnetin glycosides than their corresponding data of apical to basolateral, whereas for isorhamnetin aglycone this value was two-fold lower (Figure 2a). Similar results were observed for chrysin, which had a permeability from the basolateral to the apical direction two-fold higher than the apical to the basolateral data [36]. The efflux ratios [Papp_(BL-AP)_/Papp_(AP-BL)_] across the Caco-2 cell monolayers were higher for isorhamnetin triglycosides IGRR (5.9) and IGRP (5.2), followed by isorhamnetin diglycosides, isorhamnetin-3-*O*-glucosyl-pentoside [IGP], and isorhamnetin-3-*O*-glucosyl-rhamnoside [IGR] with 3.0 and 3.1, respectively (Figure 2b). On the other hand, the efflux ratio for isorhamnetin aglycone was 0.6, similar to the value previously reported in the Caco-2 cell monolayer [23,35].

### 2.4. Validation of the Method to Measure Isorhamnetin in Plasma 

Calibration curves with isorhamnetin and quercetin (used as an internal standard) were prepared at concentrations from 1 to 100 µg/mL. There was a good correlation between the peak area and isorhamnetin or quercetin concentration in the test ranges. The regression equations were *y* = 21.905*x* − 3.543 (*R*^2^: 0.9990) for isorhamnetin and *y* = 34.922*x* − 20.218 (*R*^2^: 0.9997) for quercetin, where *x* was the standard concentration and *y* was the peak area. Based on a signal-to-noise ratio (*S*/*N* = 10), the lower limit of quantification for isorhamnetin and quercetin was 1 µg/mL.

#### Recovery of Isorhamnetin Aglycone after Administration of *O. ficus-indica* Extract

**Figure 3:**
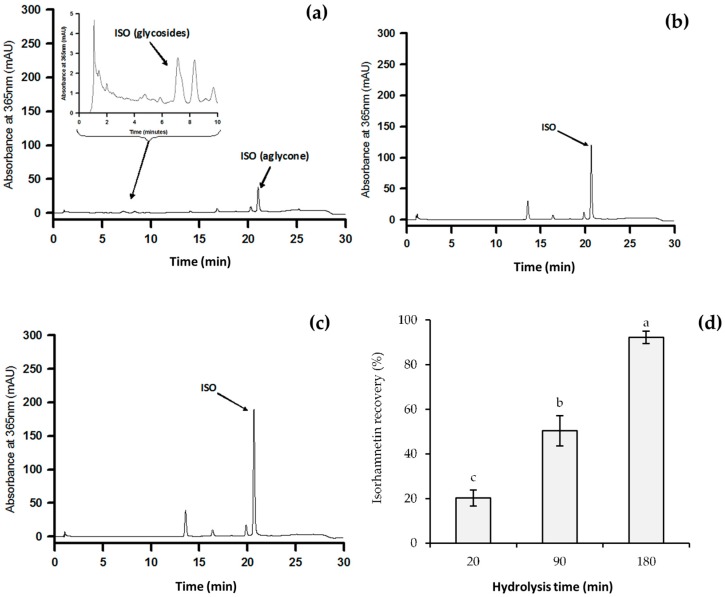
Chromatograms showing isorhamnetin release from isorhamnetin glycosides contained in *O. ficus-indica* extract, using (**a**) 20 min; (**b**) 90 min; or (**c**) 180 min of acid hydrolysis; (**d**) isorhamnetin recovery percentages obtained after 20, 90, or 180 min of acid hydrolysis. Different letters indicate significant differences among different isorhamnetin glycosides.

Isorhamnetin recovery percentages from plasma samples increased with hydrolysis time (Figure 2). Using 20 min of acid hydrolysis resulted in a non-complete release of isorhamnetin from glycosides (Figure 3a). Isorhamnetin glycosides were not detected in samples at 90 or 180 min of acid hydrolysis (Figure 3b,c). The best isorhamnetin recovery of 92.2% was found when acid hydrolysis was performed for 180 min (Figure 3d).

### 2.5. Stability of Isorhamnetin Glycosides in Plasma

**Figure 4:**
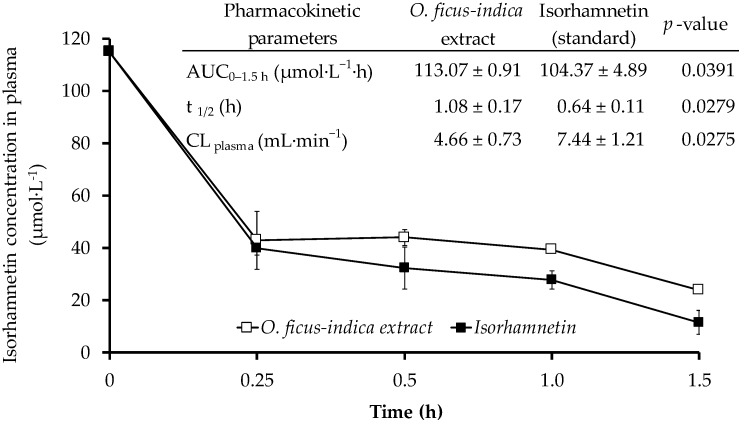
Isorhamnetin concentration (µmol·L^−1^) in rat plasma and pharmacokinetic parameters after an intravenous dose (2 mg/kg) of an *O. ficus-indica* extract or isorhamnetin standard.

Using the best conditions described above, the mean recovery percentage for quercetin (internal standard added to plasma samples) was 94.3% ± 3.9 (*n* = 24). Although the same intravenous dose (2 mg/kg) of an *O. ficus-indica* extract and an isorhamnetin pure standard (aglycone) were used, it was observed that the area under the curve (AUC) of isorhamnetin was higher for the ones that received an *O. ficus-indica* extract (AUC_0–1.5 h_: 113.07 ± 0.91 µmol·L^−1^·h) than for their counterparts that received isorhamnetin as aglycone (pure standard) (AUC_0–1.5 h_: 104.37 ± 4.89 µmol·L^−1^·h) (Figure 4). As depicted in Figure 4, the *O. ficus-indica* extract administration resulted in a 63% decrease clearance of isorhamnetin from plasma (*p* < 0.05), resulting in a 59% increase in half-life (*p* < 0.05).

## 3. Discussion

### 3.1. Bioaccessibility and Intestinal Permeability of Isorhamnetin Glycosides

Digestion process alters and even degrades the phenolics structure, affecting their stability, bioaccessibility and possible beneficial effects. The isorhamnetin recovery percentages after oral and gastric phase digestion were lower than those observed for its glycosides, providing evidence of glycosides resistance to acid degradation in the stomach. In contrast, the recovery of isorhamnetin glycosides decrease after the intestinal digestion phase suggesting that structural alterations occurred under alkaline conditions. Similarly, Tenore et al. [37] observed a decrease of 18.6 % in quercetin-3-glucosyl-rhamnoside levels after the intestinal digestion phase when compared with those of the gastric step. Rodríguez-Roque et al. [38] observed a significant decrease of isoflavones glycosides after the intestinal digestion phase compared to their recovery after the gastric step. The recovery decrease percentages of dadzein glycoside and genistein glycoside were 17.5% and 18.3%, respectively.

The sugar moiety attached to flavonol did not significantly affect the recovery, as it was previously observed by Ortega et al. [39] who obtained recoveries of 51.5% and 44.2% for myricetin-rhamnoside and myricetin-glucoside respectively, after a simulated digestion process. Likewise, Ydjedd et al. [40] reported that after intestinal digestion quercetin rhamnoside and myricetin rhamnoside showed recoveries of 47.9% and 40.2%, respectively. Moreover, glycosylation plays a protective role during the digestion process. In accordance with our results, Juániz et al. [41] showed that quercetin diglycosides (quercetin-glucosyl-rhamnoside and quercetin-sambubiosidyl-rhamnoside) had similar recovery indexes to quercetin glucoside after the digestion process.

Regarding isorhamnetin aglycone, it showed higher recovery percentages after intestinal digestion since its solubility was improved under alkaline conditions. Chandrasekara and Shahidi [42] observed a similar behavior after a simulated digestion process of different varieties of cooked millet grains. The recovery of flavonoids aglycones after the intestinal digestion phase was about 1.9 to 5.6-fold higher than the gastric phase. Likewise, Weathers et al. [43] demonstrated that the recovery of free flavonoids extracted from *Artemisia annua* leaves was 26.0% higher in the intestinal digestion phase than their content at the end of the gastric digestion phase. 

After overcoming the digestion stage, the compounds will encounter a new challenge: to cross through the biological membranes. Then they reach the systemic circulation and therefore exert a specific biological activity. In this study, the apparent permeability (Papp) of the compounds across a Caco-2/HT-29 monolayer was evaluated as a parameter to predict their bioavailability in vivo. Due to their difference in hydrophilicity that hinders their entry through the cell membrane, isorhamnetin glycosides showed lower values of apparent permeability coefficient from apical to basolateral direction (Papp_(AP-BL)_) than aglycone. On the other hand, the Papp_(AP-BL)_ values obtained with the co-culture system conformed by Caco-2/HT-29 (75:25) were higher compared with reported Papp_(AP-BL)_ values where a membrane of Caco-2 cells was used. The Caco-2 cells overexpress P-gp transporters that mediate the xenobiotic efflux out from the cell. In a co-culture of Caco-2/HT-29 the number of P-gp transporters decreased, favoring the permeability of the compounds from the apical to the basolateral side. Additionally, this model of co-culture simulates more closely the permeability of the human intestinal barrier with mucus production [44,45]. It has been reported that flavonoid glycosides showed an improved permeability rate through the mucus layer to reach the small intestines [46]. Moreover, the Papp value of flavonoids can be affected by the presence of other components that occur in natural extracts. In this way, Xie et al. [47] reported that the Papp value of isorhamnetin when tested alone was 1.61 × 10^−6^ cm/s but, in combination with kaempferol or quercetin, this Papp value increased 1.33 and 2.38-fold when tested in a Caco-2 monolayer.

On the other hand, the glycosylation profile affects the permeability of flavonoid glycosides as observed for the triglycoside isorhamnetin-3-*O*-glucosyl-rhamnosyl-rhamnoside (IGRR). The presence of an additional rhamnose moiety affected the ability of IGRR to cross the monolayer. De Araújo et al. [48] demonstrated that quercetin-3-*O*-glucoside, obtained from the enzymatic cleavage of the rhamnose moiety of quercetin-3-*O*-rhamnosyl-glucoside, showed higher antioxidant and antiproliferative effects than quercetin and rutin on different cancer cell lines. Additionally, Nielsen et al. [49] showed that removing the rhamnose moiety from hesperetin-7-glucosyl-rhamnoside improved the bioavailability by two-fold, probably the resulting flavonoid glycoside (hesperetin-7-glucoside) had a different absorption site from the colon to the small intestine. Likewise, the glycosylation pattern of flavonoids affects the uptake and efflux mechanisms of cells, favoring or limiting their bioavailability. Duan et al. [24] found that the efflux ratios of quercetin monoglycosides were different depending on the sugar moiety substitution. Quercetin-3-galactoside showed a higher efflux ratio than quercetin-3-glucoside. Since efflux ratios lower than 1.0 are indicative of the uptake mechanisms that facilitate bioavailability, therefore this data indicates that isorhamnetin aglycone could be more bioavailable than their corresponding glycosides [24].

It is important to point out that in addition to the observed differences in the absorption of isorhamnetin glycosides, their interaction with intestinal microbiota must be taken into consideration. In some cases, deglycosylation occurs differently depending on the microbiota of the host but, also, flavonoid glycosides may have a prebiotic effect and favor the growth of less dominant bacteria in the gut [50]. Interestingly, it has been reported that antibiotic treated mice had higher flavonoids excretion and therefore they were not further catabolized as in control mice [51]. The glycosylation profile is a determinant for the absorption and metabolism of flavonoids by gut microbiota due to the specificity of the enzymes responsible for the hydrolysis of the glycosidic bond [52,53]. Therefore, the gut microbiota of the individuals included in an in vivo study should be analyzed along with the flavonoid bioavailability and metabolism.

### 3.2. Stability of Isorhamnetin Glycosides in Plasma

In humans, quercetin glucosides (AUC_0–24 h_: 9.7 ± 6.9 µg·mL^−1^·h) remained longer in the organism than quercetin rutinosides (AUC_0–24 h_: 3.8 ± 3.9 µg·mL^−1^·h) and quercetin as aglycone (AUC_0–24 h_: 2.5 ± 2.2 µg·mL^−1^·h) [54]. Wang et al. [55] detected higher concentrations (10–15 ng·mL^−1^) of quercetin-3-galactoside in plasma after the ingestion of cranberry juice, indicating the enhanced bioavailability of the intact flavonol glycoside. Petersen et al. [56] reported that quercetin from an apple peel extract showed a higher AUC_0–1440 min_ (87 ± 27 µmol·L^−1^·min) value than that after the administration of quercetin aglycone (62 ± 12 µmol·L^−1^·min). Sandhu et al. [57] also demonstrated that the number of sugar moieties significantly affected the bioavailability and pharmacokinetic of flavonoid glycosides. These results indicated that isorhamnetin glycosides were more stable and therefore remained longer in plasma than isorhamnetin as aglycone.

## 4. Materials and Methods

### 4.1. Chemicals and Reagents

Isorhamnetin standard (PubChem CID: 5281654) was purchased from Indofine Chemical Co. Inc. (Hillsborough, NJ, USA). Dulbecco’s Modified Eagle’s Medium (DEMEM-F12) was obtained from Thermo Fisher Scientific (Waltham, MA, USA). The α-amilase from porcine pancreas (15 Units/mg solid), porcine pepsin (250 Units/mg solid), methasulfonic acid, HEPES sodium salt, lucifer yellow, quercetin standard, and formic acid solution HPLC grade were purchased from Sigma-Aldrich (St. Louis, MO, USA). Phosphate saline solution (PBS) pH 7.4 (1×), Hank’s balanced salt solution (HBSS), fetal bovine serum (FBS), penicillin (100,000 Unit/mL)/streptomycin (10,000 µg/mL), and trypsin-EDTA 0.25% (*w*/*v*) were acquired from Gibco Laboratories (Grand Island, NY, USA). HPLC grade water and methanol were obtained from VWR International LLC (West Chester, PA, USA).

### 4.2. Preparation of O. ficus-indica (L.) Extract

The *O. ficus-indica* (L.) plant used was harvested in the region of Montemorelos (25°11′ north, 99°50′ west and 342 m above the sea level), Nuevo León, México, and the taxonomic identification was done at the School of Agronomy of Universidad Autónoma de Nuevo León (UANL), México. *O. ficus-indica* pads (7 months of age) were processed, as reported by Antunes-Ricardo et al. [1], to obtain a flour (particle size < 180 µm), which was packaged in dark bags and stored at −20 °C until extractions. *O. ficus-indica* extract was obtained following the procedure described by Rodríguez-Rodríguez et al. [10]. *O. ficus-indica* flour was extracted with methanol 80% (1:10 *w*/*v*), stirred at 200 rpm for 12 h. Supernatant was filtered (Whatman No. 1) and concentrated during 40 min at 50 °C and 85 mbar (Flawil, Switzerland). Thereafter, contents of isorhamnetin glycosides in the *O. ficus-indica* extract were enriched by solid phase extraction (SPE) using a C_18_ column (Strata C18-E; 55μm, 70A). The faction which eluted from the C_18_ column with 100% methanol was recovered and taken to dryness, then lyophilized and stored at −20 °C until use.

### 4.3. Analysis of Isorhamnetin Glycosides by High Performance Liquid Chromatography (HPLC)

Chromatographic analysis of isorhamnetin and its glycosides in the *O. ficus-indica* extract was performed according to the method and conditions reported by Rodríguez-Rodríguez et al. [10]. Separation was performed in a Zorbax with Eclipse Extra Dense Bonding (XDB) C18 4.6 mm × 150 mm, 5 µm column (Agilent Technologies, Santa Clara, CA, USA) with a flow of 0.45 mL/min. The mobile phase consisted of (A) HPLC-grade water with 0.1% of formic acid and (B) HPLC-grade methanol. The gradient started with 35% of B for 5 min, increasing to 60% in 15 min, and in the next 5 min the percentage of B changed to 90%. Chromatograms were obtained at 365 nm. Standard curves for isorhamnetin and quercetin were used. Isorhamnetin glycosides were quantified as isorhamnetin equivalents. Identification of isorhamnetin glycosides was performed by Liquid chromatography coupled with time-of-flight mass spectrometry [LC/MS-TOF] (Agilent Technologies, Santa Clara, CA, USA) according to the method described by Santos-Zea et al. [5].

### 4.4. Simulated In Vitro Digestion

The simulated in vitro digestion model was performed based on the methodology described by Garrett et al. [58], with some modifications. Isorhamnetin aglycones and *O. ficus-indica* extract were subjected to digestion in triplicate. A three-step procedure was applied to simulate the digestive processes in the mouth, stomach, and small intestine. The digestion started with an amount of extract that contained an equivalent of 10 mg of isorhamnetin (60 mg) or 10 mg of isorhamnetin standard. An α-amylase solution (75 Units/mL) was added to 20 mL of solution, adjusted to pH 6.9, which was incubated for 10 min. This mixture was shaken using a VWR^®^ Incubating orbital shaker (Thorofare, NJ, USA) for 10 min at 37 °C and 55 rpm. The gastric digestion started by adjusting the pH to 2 with 1 M HCl and, after, 2 mL of porcine-pepsin solution was added (40 mg/mL in 0.1 M HCl). This mixture was shaken in an incubator for 1 h at 37 °C and 95 rpm. For the intestinal digestion, the solution was previously adjusted to pH 5.3 with 0.9 M NaHCO_3_ and then a mixture of bile salts and pancreatin (6 mL containing porcine-pancreatin (2 mg/mL), and bile salts (12 mg/mL) dissolved in a NaHCO_3_ solution (100 mM), were added. Concentrations of pancreatin and bile extract in the final solution were 0.4 and 2.4 mg/mL, respectively. Then, the solution was adjusted to pH 7.5 by adding 1N NaOH. The mixture was shaken in an incubator for 2 h at 37 °C and 95 rpm. The enzyme reaction was inactivated by placing the sample in a hot water bath (80 °C for 2 min). At the end of the process, the digested samples were centrifuged at 13,000× *g* and 4 °C for 5 min (Thermo Scientific SL 16R centrifuge, Osterode, Germany). The resulting supernatant, which was considered the bioaccessible or absorbable fraction, was recovered. The recovery percentage of each compound was calculated based on the concentration in the supernatant recovered after each phase (oral, gastric, and intestinal) of the in vitro digestion. This fraction was also tested for permeability and potential bioavailability of the isorhamnetin glycosides present in the *O. ficus-indica* extract.

### 4.5. Cell Culture and Permeability Experiments

Caco-2 and HT-29 cell lines were obtained from ATCC (American Tissue Culture Collection, Rockville, MD, USA). Cells were cultured in DMEM-F12 supplemented with 10% fetal bovine serum and were maintained at 37 °C in 5% CO_2_. Before the start of the permeability experiments, the cytotoxicity of isorhamnetin and the *O. ficus-indica* extract on Caco-2/HT-29 (75:25) co-culture were tested to ensure that the viability of the cells was over 90%. Permeability experiments were performed according to Xie et al. [18] and Hubatsch et al. [59] with slight modifications. Caco-2 and HT-29 co-culture cell monolayer (75:25) [44] were seeded on 6-well transwell plates, with inserts having a polycarbonate membrane with a surface area of 4.71 cm^2^ and a pore diameter of 0.4 µm (Costar, Corning, NY, USA) at a final seeding density of 1 × 10^6^ cells/insert, cultured for 21 days. Caco-2 cell monolayers were washed with warm HBSS medium (pH 7.4) and then digested samples of isorhamnetin aglycone or *O. ficus-indica* extract were added to the apical side (AP: 1.5 mL), whereas the receiving compartment or basolateral side (BL: 2.5 mL) contained the corresponding volume of HBSS. Plates with transwell inserts were incubated at 37 °C in an orbital shaking incubator at 100 rpm. Aliquots of 1 mL were withdrawn from both compartments at 15, 30, 60 and 120 min, and fresh HBSS was added to replace the withdrawn volume. Samples were immediately frozen and preserved at −20 °C until HPLC analysis. All experiments were performed in triplicate.

The apparent permeability coefficient (Papp) was calculated from the cumulative amount permeated versus the time profile according the following Equation (1):

Papp = (dQ/dt) × (V/A × C_0_) (1)

where dQ/dt is the change in drug concentration in the receiver solution (µM/s), *V* represents the volume of the solution in the receiving compartment (mL), A denotes the membrane surface area (cm^2^), and C_0_ is the initial concentration in the donor compartment (µM).

The integrity of the monolayers was measured at the beginning and the end of the experiment using the paracellular permeability of lucifer yellow (LY). Fluorescence was measured at 530 nm (emission) and 485 nm (excitation) using a microplate reader (Synergy HT, BioTek, Winooski, VM). The data from membranes displaying an LY apparent permeability coefficient (Papp) > 1 × 10^−6^ cm/s were excluded.

### 4.6. Validation of the Method to Measure Isorhamnetin in Plasma

The high-performance liquid chromatography (HPLC) method was validated for the linearity and sensitivity. Blank plasma samples (100 µL) were spiked with known quantities (1.0, 5.0, 10, 20, and 100 µg/mL) of isorhamnetin or quercetin standards. Briefly, 0.6 mL of 2 M HCl was added to each plasma sample and mixed in vortex for 1 min. Then, samples were hydrolyzed at 80 °C during 180 min; afterwards, 1 mL of 100% methanol was added to each hydrolyzed sample and vortexed for 1 min in order to enhance protein precipitation. The mixtures were centrifuged (2000× *g*, 10 min at 4 °C) and then mixed (10:1, *v*/*v*) with 0.5 M acetic acid, which contained 2 mg of vitamin C per milliliter, to prevent the loss of flavonols, because plasma pH increases with time [60]. The supernatants were recovered and evaporated to dryness. Residues were reconstituted with 150 µL of methanol, filtered and analyzed with HPLC-PDA. Peak areas of isorhamnetin and quercetin were plotted versus its concentrations to construct the calibration curves. The linearity of the calibration curves was calculated and correlation coefficients (*R*^2^) of 0.99 or better were selected. The lower limit of quantification was defined as the concentration with an accuracy of 80–120% and precision (% coefficient of variation ) < 20%.

#### Recovery of Isorhamnetin Aglycone after Administration of *O. ficus-indica* Extract

Rat plasma was enriched with known quantities (100 µg/mL) of isorhamnetin standard or isorhamnetin equivalents from *O. ficus-indica* extract. Samples were processed as previously described (see Section 4.6) using different hydrolysis times (20, 90, or 180 min). The recovery percentage of isorhamnetin (as aglycone) was calculated by comparing the peak areas of the isorhamnetin standard (A) with those of the isorhamnetin equivalents added to the plasma as *O. ficus-indica* extract (B) according the following Equation (2):

%Recovery = (A/B) × 100
(2)

### 4.7. Stability of Isorhamnetin Glycosides in Plasma

#### 4.7.1. Animal Care

The study was approved by the Institutional Committee on the Care and Use of Experimental Animals (CICUAL) at the Tecnologico de Monterrey (Protocol number 2011–021; 3 February 2012). Animals were obtained from the Center of Experimental Animals at the School of Medicine and Health Science (Monterrey, Mexico) and kept in cages in a room with 12 h light/dark cycles at 22 °C and relative humidity (50 ± 5%) with free access to diet and water. Animals were acclimatized for 10 days prior to the experiments.

#### 4.7.2. Pharmacokinetics Parameters Determination

Twenty-seven male Wistar rats, weighing approximately 200 g (eight weeks old), were divided in nine groups of three animals each. One group was taken as a control and received only a saline solution through the caudal vein. Four groups received an intravenous dose (2 mg/kg) of the *O. ficus-indica* extract and the last four groups received the commercial isorhamnetin standard. The dosage was determined based on results previously reported by Antunes-Ricardo et al. [1]. Animals were anaesthetized with ketamine/xilazyne (60 mg/kg; 10 mg/kg), and then blood was extracted by intracardiac puncture at different sampling times (0.25, 0.5, 1 and 1.5 h). This procedure was randomized to avoid any bias results. After blood extraction, the animals were sacrificed by cervical dislocation. Plasma was obtained as previously described (see Section 4.6) and the samples were stored at −20 °C until analysis.

Plasma samples were spiked with 25 µL of quercetin (30 µg/mL) as an internal standard. Then, plasma samples were submitted to an acid hydrolysis with 2 M HCl for 180 min at 80 °C. Then, samples were extracted with 100% methanol (10:1, *v*/*v*), centrifuged, and the resulting supernatants were quantified by HPLC-PDA. The area under the concentration-time curve (AUC_0−1.5 h_) was calculated based on the linear trapezoidal method. The elimination half-life (t_1/2_) was determined by linear regression of the terminal portion of the plasma concentration-time data. The plasma clearance (CL_plasma_) was calculated by multiplying the volume of distribution, Vd, by the elimination constant, Kel, according to the following Equation (3):

CL_plasma_: Vd × Kel (3)

### 4.8. Statistical Analysis

Experiments were performed at least by triplicate and the results were analyzed with JMP 13.0 software (SAS Institute Inc., Cary, NC, USA) using ANOVA followed by Tukey’s HSD tests. For each data set, *p* < 0.05 was considered statistically significant.

## 5. Conclusions

Glycosylation protected isorhamnetin from its degradation during simulated digestion, but affected the permeability through the Caco-2/HT-29 monolayer. Based on our results, the diglycosides had better permeability than triglycosides, independent of their sugar substitution. Therefore, biotechnological strategies should be developed to partially release isorhamnetin mono- and diglycosides from *O. ficus-indica* to improve their bioavailability and avoid the release of their aglycone, since they would be more easily cleared from the circulatory system. Likewise, the conjugation of therapeutic molecules with these compounds would prolong their biological half-life and, therefore, their biological effects.

## Figures and Tables

**Table 1:** Comparison of recovery percentage (%) of isorhamnetin glycosides (IGRR, IGRP, IGP, IGR) from raw *O. ficus-indica* extract and isorhamnetin standard after human-simulated digestion.

Isorhamnetin Glycosides	Digestion Phase		
	Oral	Gastric	Intestinal
IGRR	99.1 ± 3.8 ^A,a^	96.0 ± 4.4 ^A,a^	79.5 ± 1.8 ^A,b^
IGRP	93.8 ± 5.2 ^A,a^	96.2 ± 0.4 ^A,a^	80.3 ± 1.9 ^A,b^
IGP	96.6 ± 4.7 ^A,a^	93.5 ± 3.3 ^A,a^	73.3 ± 3.5 ^A,b^
IGR	99.6 ± 3.8 ^A,a^	96.8 ± 0.9 ^A,a^	81.0 ± 4.6 ^A,b^
I	35.0 ± 2.4 ^B,b^	49.0 ± 3.4 ^B,b^	74.3 ± 7.4 ^A,a^

IGRR: isorhamnetin-3-*O*-glucosyl-rhamnosyl-rhamnoside; IGRP: isorhamnetin-3-*O*-glucosyl-rhamnosyl-pentoside; IGP: isorhamnetin-3-*O*-glucosyl-pentoside; IGR: isorhamnetin-3-*O*-glucosyl-rhamnoside; I: isorhamnetin. ^A,B^ Significant differences of the recovery percentage between the isorhamnetin glycosides in each digestion phase. ^a,b^ Significant differences between the values of recovery percentage of each isorhamnetin glycosides between the different digestion phases.

## Abbreviations

AUC	Area under the concentration-time curve
CL	Plasma clearance
Cmax	Maximum plasma concentration
IGRR	Isorhamnetin-3-*O*-glucosyl-rhamnosyl-rhamnoside
IGRP	Isorhamnetin-3-*O*-glucosyl-rhamnosyl-pentoside
IGP	Isorhamnetin-3-*O*-glucosyl-pentoside
IGR	Isorhamnetin-3-*O*-glucosylrhamnoside
I	Isorhamnetin
Papp	Apparent permeability coefficient
Papp_(AP-BL)_	Apparent permeability coefficients from apical to basolateral direction
t_1/2_	Elimination half-life
